# Effect of Aquatic Exercise Training on Aortic Hemodynamics in Middle-Aged and Elderly Adults

**DOI:** 10.3389/fcvm.2021.770519

**Published:** 2021-11-02

**Authors:** Marina Fukuie, Takayuki Yamabe, Daisuke Hoshi, Tatsuya Hashitomi, Yosuke Nomura, Jun Sugawara

**Affiliations:** ^1^Doctoral Program in Sports Medicine, Graduate School of Comprehensive Human Sciences, University of Tsukuba, Tsukuba, Japan; ^2^Human Informatics and Interaction Research Institute, National Institute of Advanced Industrial Science and Technology, Tsukuba, Japan; ^3^Tsukuba Aqualife Laboratory, Tsukuba, Japan; ^4^Faculty of Health and Sports Sciences, University of Tsukuba, Tsukuba, Japan

**Keywords:** aquatic exercise, aortic blood pressure, reflection wave, Windkessel function, polar coordinate description

## Abstract

Aquatic exercise is an attractive form of exercise that utilizes the various properties of water to improve physical health, including arterial stiffness. However, it is unclear whether regular head-out aquatic exercise affects aortic hemodynamics, the emerging risk factors for future cardiovascular disease. The purpose of this study was to investigate whether head-out aquatic exercise training improves aortic hemodynamics in middle-aged and elderly people. In addition, to shed light on the underlying mechanisms, we determined the contribution of change in arterial stiffness to the hypothesized changes in aortic hemodynamics. Twenty-three middle-aged and elderly subjects (62 ± 9 years) underwent a weekly aquatic exercise course for 15 weeks. Aortic hemodynamics were evaluated by pulse wave analysis *via* the general transfer function method. Using a polar coordinate description, companion metrics of aortic pulse pressure (PPC = √{(systolic blood pressure)^2^ + (diastolic blood pressure)^2^}) and augmentation index (AIxC = √{(augmentation pressure)^2^ + (pulse pressure)^2^}) were calculated as measures of arterial load. Brachial-ankle (baPWV, reflecting stiffness of the abdominal aorta and leg artery) and heart-ankle (haPWV, reflecting stiffness of the whole aortic and leg artery) pulse wave velocities were also measured. The rate of participation in the aquatic training program was 83.5 ± 13.0%. Aortic systolic blood pressure, pulse pressure, PPC, AIxC, baPWV, and haPWV decreased after the training (*P* < 0.05 for all), whereas augmentation index remained unchanged. Changes in aortic SBP were correlated with changes in haPWV (*r* = 0.613, *P* = 0.002) but not baPWV (*r* = 0.296, *P* = 0.170). These findings suggest that head-out aquatic exercise training may improve aortic hemodynamics in middle-aged and elderly people, with the particular benefits for reducing aortic SBP which is associated with proximal aortic stiffness.

## Introduction

The advancing age is the major risk factor for cardiovascular diseases (CVD), and this is attributable in part to stiffening of large elastic arteries (1). Central arterial stiffening leads to the augmented wave reflection which pre-maturely return during early systole and thus increases aortic systolic blood pressure (SBP), pulse pressure (PP), and augmentation index (AIx). These hemodynamic characteristics could be independent predictors of future cardiovascular events and all-cause mortality (2).

Regular aerobic exercise is a recommended lifestyle for preventing CVD and risk factors (3, 4). Particularly, aquatic exercise (e.g., swimming and head-out aquatic exercise) has less stress on joints and lower risk of falling due to buoyancy and water resistance, and it is performed by a wide range of populations such as overweight and obese individuals (5, 6), the elderly people (7), and patients with osteoarthritis (8). Several intervention studies demonstrated that swimming exercise training improved arterial stiffness and aortic hemodynamics (8–12). On the other hand, we (13) and others (14, 15) showed head-out aquatic exercise training-induced favorable reduction in arterial stiffness, while the influence on aortic hemodynamics remained fully unknown.

**Figure 1:**
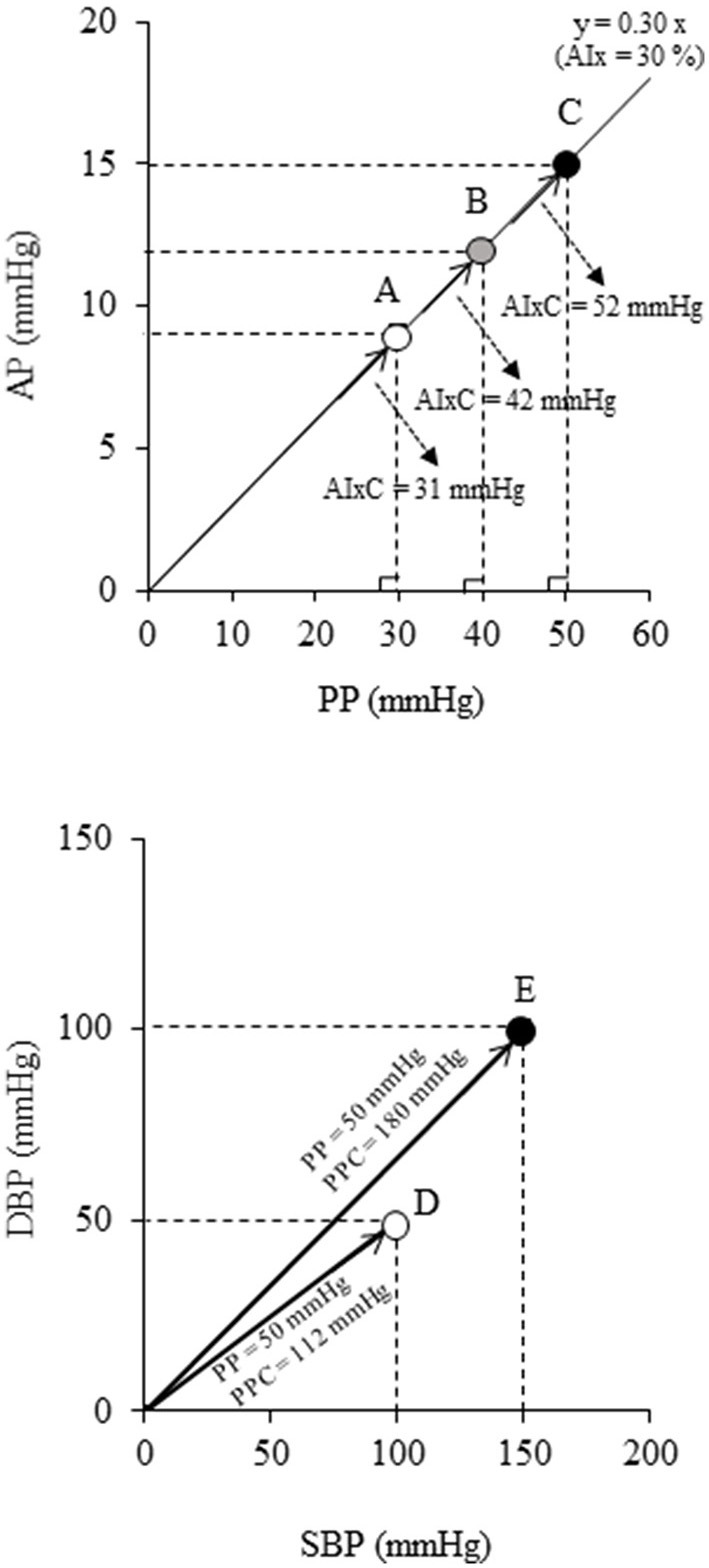
Schematic diagrams indicating the limitation of the conventional ratio-based and difference-based metrics: **(Top)** augmentation index (AIx) and **(Bottom)** pulse pressure (PP). These indices have various physiological loads even at the same values (e.g., points A-C all have AIx = 30%, points D-E both have PP = 50 mmHg). Companions of AIx (AIxC = √{AP^2^ + PP^2^}) and PP (PPC = √{SBP^2^ + DBP^2^}) are alternative data representation obtained by the polar coordinate description which permits more precise characterization of individuals (16).

Recently, there is a gaining recognition about the limitations of using dimensionless ratio-based metrics in cardiovascular medicine such as AIx. Indices based on ratios may not sufficiently reflect information about their constituents. As shown in the top panel in Figure 1, although points A, B, and C have different magnitudes of wave reflection from the periphery (i.e., augmented pressure, AP), the same values of AIx are obtained for the corresponding aortic PP values. After calculating AIx, the physiological units of AP and PP are canceled out, which results in a dimensionless number that no longer characterizes the unique properties of cardiac load. To overcome such simplification, polar coordinate description has recently been proposed as an alternative method (16). Using this method, the ratio can be presented in the pressure domain using a hypotenuse *via* the Pythagorean theorem as the “companion” of AIx (AIxC) (17). Likewise, the “companion” of PP (PPC) can also be calculated by the Pythagorean theorem. In the bottom panel of Figure 1, points D and E have different PPC for the same PP (i.e., 50 mmHg). Thus, the polar coordinate description may offer a clue to the physiologically relevant interpretation without need for additional measurement.

The purpose of this study was to investigate the hypothesis that head-out aquatic exercise training improves aortic hemodynamics in middle-aged and elderly people. Combining the traditional and newly described companion metrics (i.e., AIxC and PPC), we characterized changes in aortic hemodynamics comprehensively. In addition, to shed light on the underlying mechanisms, we determined the contribution of change in arterial stiffness to the hypothesized changes in aortic hemodynamics.

## Materials and Methods

### Subjects

A total of 59 individuals joined the city-operated aquatic exercise training class. Of all, 38 volunteers (10 men, 4 pre-menopausal, and 24 post-menopausal women, age range: 39–79 years) gave their written informed consent after the explanation of all potential risks and procedures of the present study. No one engaged in regular intense endurance training such as joining masters' competition. All subjects were non-smokers. No women were taking hormone replacement therapy. This study was reviewed and approved by the Institutional Research Board of the National Institute of Advanced Industrial Science and Technology (#2017-759).

### Aquatic Exercise Training Program

The exercise program consisted of 15 training sessions (once a week) (from July to October 2017). Each exercise session consisted of a 5-min warm-up (i.e., stretching) on land and 50 min of aquatic exercise in indoor pool (31.5°C of water temperature; 1.05–1.15 m of water depth), including 10 min of walking, 30 min of resistance and stepping exercise, and 10 min of stretching and relaxation (18). The last 5 min served as the cooling down on land. Based on the subjects' age, self-reported physical activity level, and disease status (based on the most recent health checkup), each subject was assigned to the moderate (9 of 23 subjects) or the low-intensity (14 of 23 subjects) exercise classes. Five subjects in the moderate-intensity exercise class and 3 subjects in the low-intensity exercise class had exercise habits before enrolling in this intervention study. None of them had strength training or water exercise within the past 6 months. Aside from exercise training intervention, all subjects were instructed not to change daily activities, including their exercise habits. An experienced instructor supervised and provided typical water-based movements (19). Participants reported on a 6–20 scale of perceived exertion scale (RPE) during training after every class.

### Measurements

**Figure 2:**
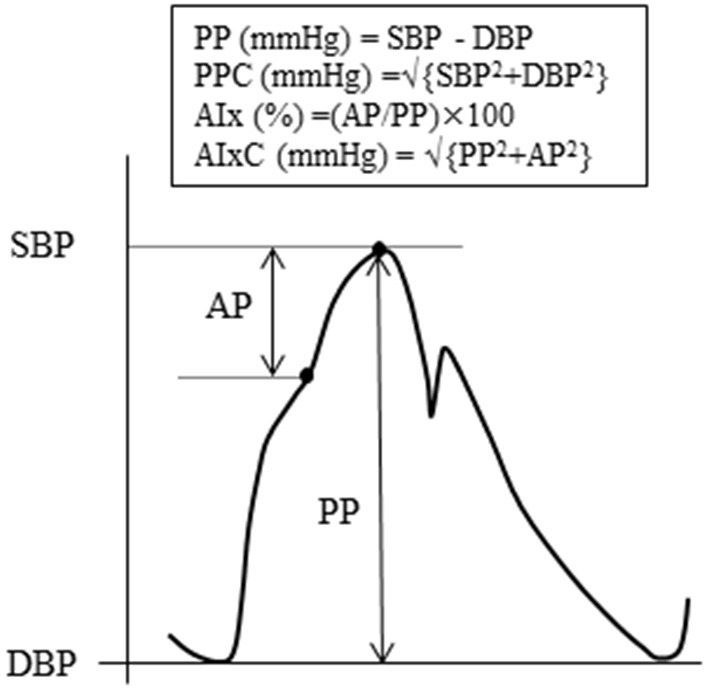
Schema of aortic pulse wave analysis. The augmentation index (AIx) is defined as the ratio of augmentation pressure (AP, from the inflection point of systolic shoulder to the systolic peak) to pulse pressure (PP, the difference between systolic and diastolic blood pressure). The companion of PP (PPC) is the square root of the sum of the squared systolic and diastolic pressures, respectively. The companion of AIx (AIxC) is the square root of the sum of the squared PP and the squared AP.

Hemodynamic variables and arterial stiffness measurement were performed after 3 h of fasting and abstinence from caffeine intake. Each subject was asked not to drink alcohol and engage in vigorous physical activity within 24 h before the experiment. Individuals with prescribed medications (hyperlipidemia: *n* = 2; diabetes mellitus: *n* = 2) were instructed to take them as usual. After 5 min of supine rest, heart rate, limb BP, heart sound, and two segments of PWV [i.e., brachial to ankle PWV (baPWV), aortic (heart) to ankle PWV (haPWV)] were measured using a semiautomatic cardiovascular screening device (Form PWV/ABI, Omron-Colin, Kyoto, Japan), as previously reported (20). Aortic BP was estimated based on the previous study (21). The brachial arterial pressure waveform was resampled at 256 Hz with data acquisition and analysis software (AcqKnowledge; BIOPAC Systems). Subsequently, the arterial waveform data were fed into the SphygmoCor Excel software (AtCor Medical, Sydney, Australia), and a generalized transfer function was applied to estimate aortic BP. Aortic BP was calibrated by brachial BP. Schema of aortic pulse wave analysis was illustrated in Figure 2. To quantify the magnitude of wave reflection from the periphery to the heart, aortic AIx was calculated as AP divided by aortic PP (22). In addition, the ratio-based measure of AIx was transformed to the pressure domain measure AIxC *via* the Pythagorean theorem (16).


$$\begin{array}{l} \text{AIxC}\left(\text{mmHg}\right)\;=\;\surd \left\{\text{A}{\text{P}}^{2}+\text{P}{\text{P}}^{2}\right\} \end{array}$$

Furthermore, the “companion” of PP was also calculated as follows.


$$\begin{array}{l} \text{PPC}\left(\text{mmHg}\right)\;=\;\surd \left\{\text{SB}{\text{P}}^{2}+\text{DB}{\text{P}}^{2}\right\} \end{array}$$

Figure 2 is the graphic representation of these aortic hemodynamics. In our pilot experiment (*n* = 5), the day-to-day reproducibility (i.e., coefficient of variation) of aortic SBP, PP, PPC, AIx, AIxC, baPWV, and haPWV was 1.2, 5.9, 1.3, 10.6, 7.2, 2.4, and 3.0%, respectively.

### Data Analysis

Exclusion criteria were (1) pre-menopause (*n* = 4) (*via* the questionnaire), (2) suspected peripheral arterial disease [ABI < 0.9 (23)] (*n* = 2), (3) antihypertensive medication (*n* = 6), (4) inability to measure heart sound (*n* = 1), and (5) outlier of baPWV (*n* = 2) which was evaluated by Smirnov-Grubbs test. A total of 23 subjects (6 men and 17 post-menopausal women) including 6 untreated hypertensives were included in statistical analysis.

### Statistic

Data are expressed as mean ± SD. Intragroup comparisons of the data obtained before and after the intervention period were performed using a paired *t*-test. Analysis of covariance (ANCOVA) was used to examine the contribution of the correlated variables (changes in haPWV and AIxC) to change in aortic SBP. Pearson's product-moment correlation was used to determine the relationship between variables of interest. Statistical significance was set a priori at *P* < 0.05 for all comparisons.

## Results

**Figure 3:**
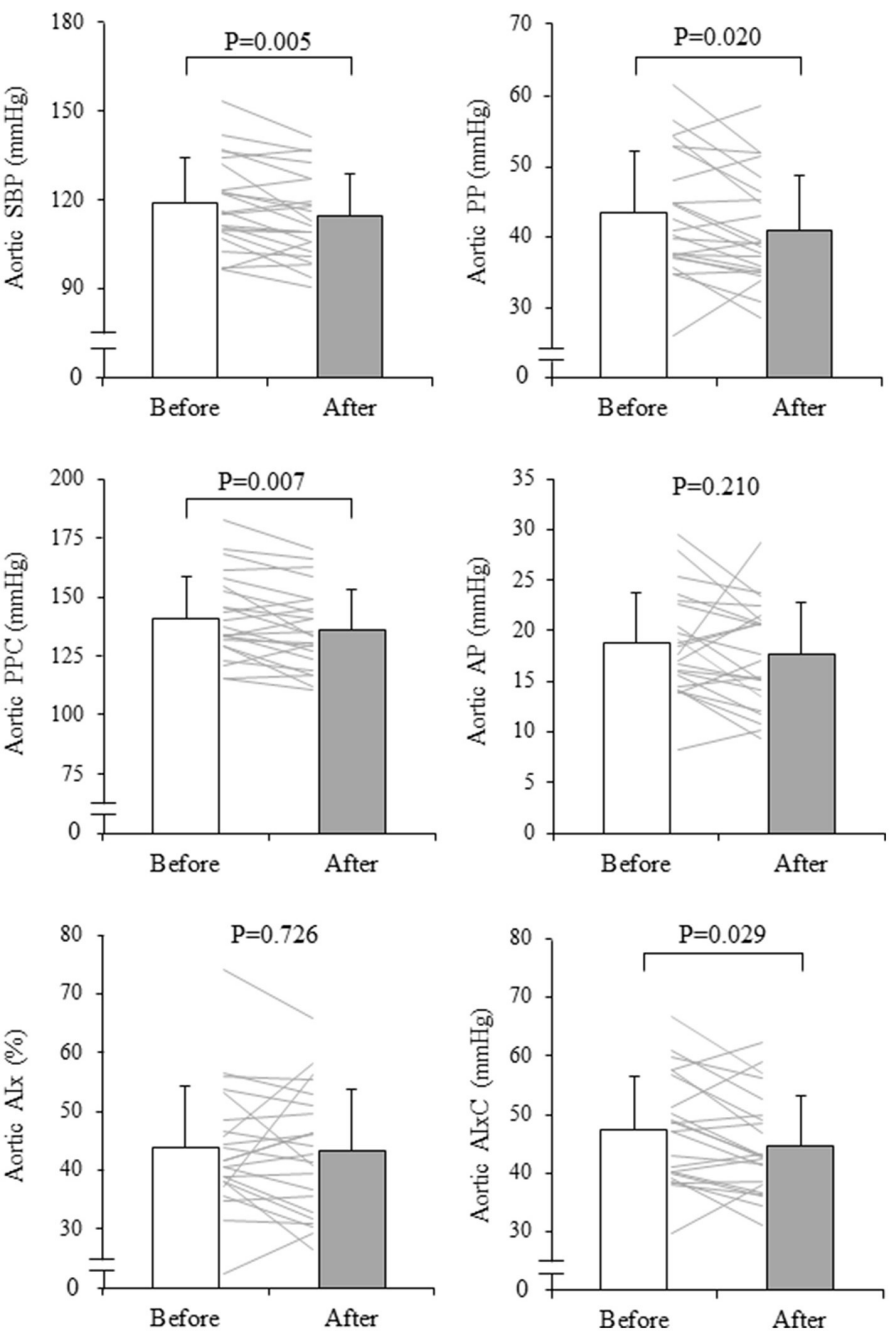
Changes in aortic systolic blood pressure (SBP), pulse pressure (PP), reflection wave (AP, augmentation pressure and AIx, augmentation index), and companion index (PPC, companion of PP and AIxC, companion of AIx) with aquatic exercise training intervention. The thin line shows the change before and after the intervention for an individual and the bars show mean ± SD. *P*-values are the results of the paired *t*-test.

The subjects analyzed had mean age of 62 ± 9 years and a height of 159.0 ± 7.4 cm. The mean RPE for all 15 sessions recorded after each class was 12.5 ± 2.0 (low-intensity class: 12.2 ± 0.6, moderate-intensity class: 12.8 ± 1.5). The rate of participation in exercise training sessions was 83.5 ± 13.0% (low-intensity class: 83.3%, moderate-intensity class: 83.7%, range 7–15 sessions). After the intervention, body weight significantly decreased (from 60.4 ± 9.7 to 58.8 ± 9.4 kg, *P* = 0.015). Heart rate and ABI did not change significantly, whereas brachial SBP, MAP, PP, baPWV, and haPWV significantly decreased after the exercise intervention (Table 1). Figure 3 depicts changes in aortic hemodynamics. Aortic SBP (from 119 ± 15 to 115 ± 14 mmHg, *P* = 0.005), PP (from 43 ± 9 to 41 ± 8 mmHg, *P* = 0.020), and PPC (from 141 ± 18 to 136 ± 17 mmHg, *P* = 0.007), decreased significantly after aquatic exercise training. AIxC decreased after exercise training intervention (from 47 ± 9 to 45 ± 8 mmHg, *P* = 0.029) although AP (from 19 ± 5 to 18 ± 5 mmHg, *P* = 0.210) and AIx (from 44 ± 10 to 43 ± 11%, *P* = 0.726) remained unchanged.

**Table 1:** Changes in heart rate, brachial blood pressure, pulse wave velocity (PWV), and ankle-brachial index (ABI) with the aquatic exercise training intervention.

**Measurements**	**Before**	**After**	***T*-test**
	**Mean ± SD**	**Mean ± SD**	***P*-value**
HR (bpm)	65 ± 8	64 ± 7	0.720
SBP (mmHg)	129 ± 17	124 ± 16	0.007
DBP (mmHg)	76 ± 10	74 ± 10	0.056
MAP (mmHg)	97 ± 13	94 ± 13	0.014
PP (mmHg)	53 ± 11	50 ± 9	0.025
baPWV (cm/s)	1,509 ± 301	1,448 ± 296	<0.001
haPWV (cm/s)	1,095 ± 141	1,072 ± 157	0.049
Right ABI (%)	110 ± 7	111 ± 6	0.246
Left ABI (%)	109 ± 7	111 ± 6	0.129

*Data are mean and SD*.

*HR, heart rate; SBP, systolic blood pressure; DBP, diastolic blood pressure; MAP, mean arterial pressure; PP, pulse pressure; baPWV, brachial to ankle PWV; haPWV, heart to ankle PWV*.

**Figure 4:**
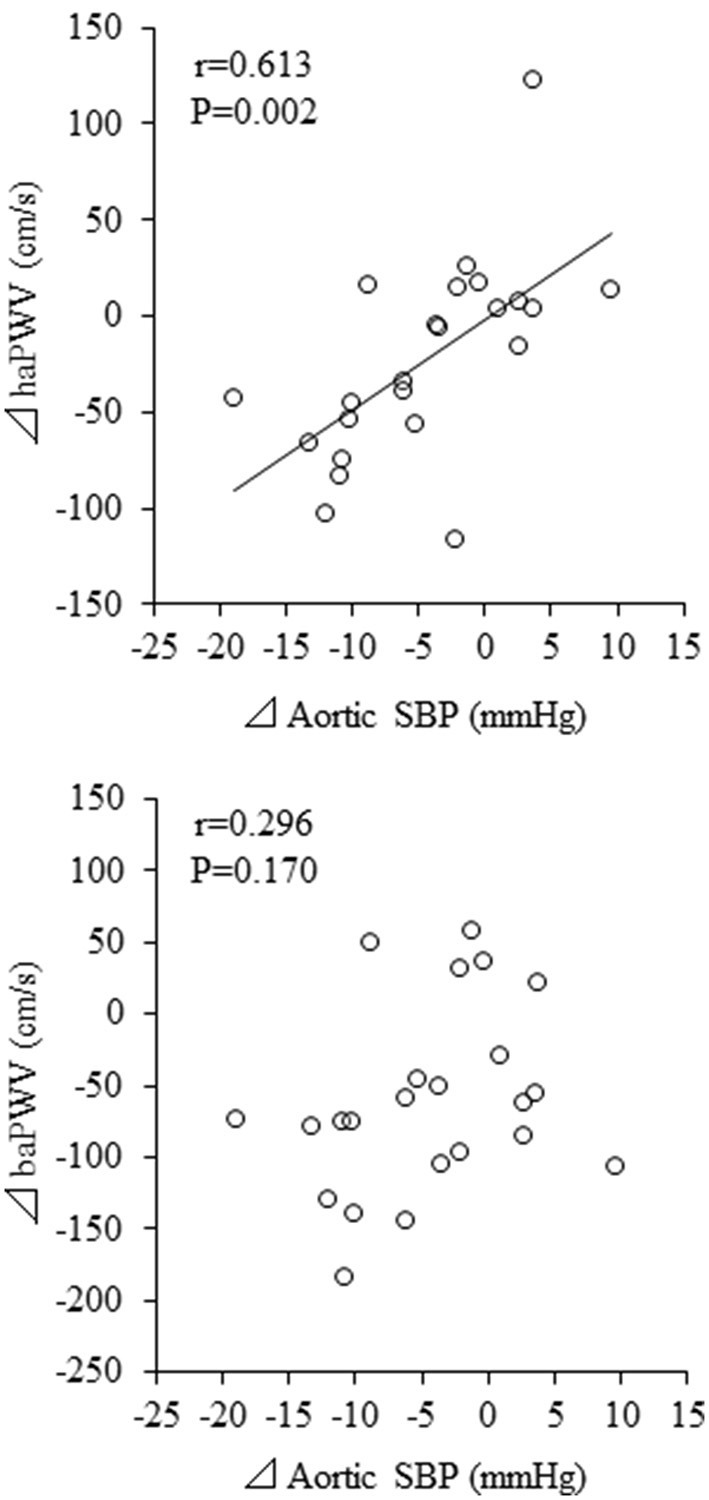
Correlations between changes in pulse wave velocity (PWV) and aortic systolic blood pressure (SBP). baPWV, brachial-ankle PWV; haPWV, heart-ankle PWV.

**Figure 5:**
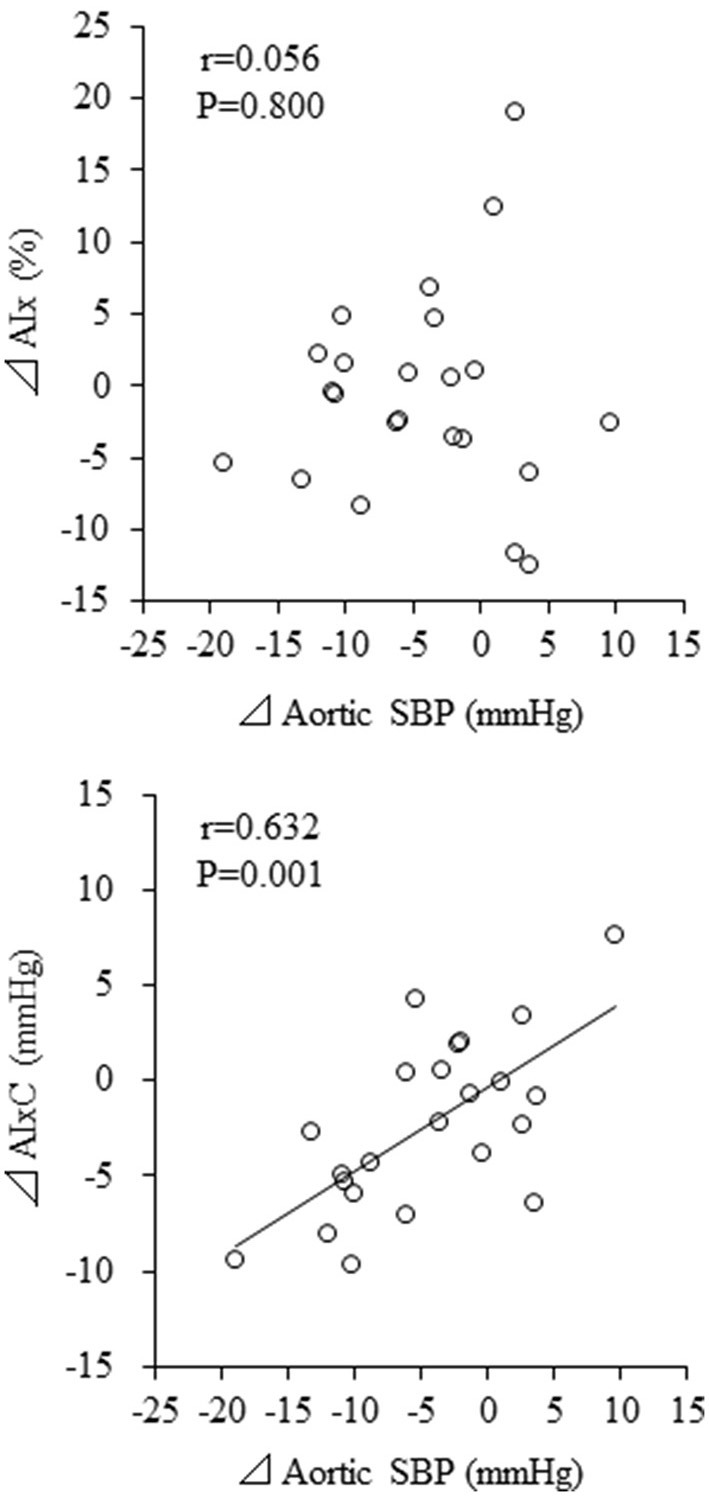
Correlations between changes in augmentation index (AIx) and aortic systolic blood pressure (SBP). AIxC, AIx-associated companion index.

Change in aortic SBP was correlated with change in haPWV (*r* = 0.613, *P* = 0.002) but not with change in baPWV (*r* = 0.296, *P* = 0.170) (Figure 4). Besides, change in aortic SBP was correlated with change in AIxC (*r* = 0.632, *P* = 0.001) but not with change in AIx (*r* = 0.056, *P* = 0.800) (Figure 5). When ANCOVA was performed with either change in haPWV or AIxC as the covariate, the change in aortic SBP was no longer statistically significant (*P* = 0.371 and *P* = 0.653, respectively). Other correlation coefficients between variables of interest were summarized in Supplementary Table 1. Changes in aortic SBP, PP, PPC, and AIxC were not correlated with either participation rate or change in body weight. Change in aortic SBP was not correlated with the baseline AoSBP (*r* = −0.350, *P* = 0.102).

## Discussion

The main findings of this study are as follows. First, short-term, head-out aquatic exercise training improve aortic hemodynamics in middle-aged and elderly subjects. Second, changes in aortic SBP were significantly associated with haPWV but not with baPWV.

To the best our knowledge, this is the first study to provide the favorable effects of head-out aquatic exercise on aortic hemodynamics, the emerging risk factors for future CV events and all-cause mortality (2). Reductions in aortic SBP and PP were only 4 mmHg and 3 mmHg. However, these changes might have pathophysiological implication because the 10 mmHg increases in aortic SBP and PP corresponded to the increasing risk for total CVD events by 8.8 and 13.7%, respectively (2). Also, it should be emphasized the low frequency (once per week) of our training regimen. Although one 60-min training session per week is typical as a city-operated aquatic exercise program, the frequency of the training is remarkably lower than those in previous studies that demonstrated the positive effect of aquatic exercise training on cardiovascular health (8–12, 14). Therefore, it is needed to examine whether the increasing frequency of the training reinforces the training effect on aortic hemodynamics.

We can only speculate the underlying mechanisms of decreased aortic SBP. The change in aortic SBP with aquatic exercise training intervention was not associated with the change in baPWV, but it was associated with the change in haPWV. Currently, baPWV has become a popular modality of arterial stiffness measurement in population-based studies because of the procedural advantage of being simple to use (i.e., only wrapping cuff-sensors around four extremities) (24). Previous studies have shown that baPWV is correlated with carotid-to-femoral PWV, the gold standard measure of aortic stiffness (25–27). baPWV may provide qualitatively similar information to those derived from central arterial stiffness, although some portions of baPWV may be determined by peripheral arterial stiffness (25). However, in the measurement of baPWV, brachial arterial pressure waveforms are used as substitutes for pressure waves in the proximal aortic site, which could not be recorded easily. Thus, the segment from the ascending aorta to the upper arm is omitted from the effective path length for baPWV, as the ascending aorta is not included in the path length for cfPWV (27). On contrary, the arterial path of haPWV includes the ascending aorta due to the use of the second heart sound, indicating the timing of the aortic valve closure (i.e., dicrotic notch of the brachial pressure wave). Therefore, the significant correlation between changes in aortic SBP and haPWV but not baPWV suggests that the proximal aortic destiffening is associated with the reduction of aortic SBP. However, the causality cannot be clarified by this study design.

The proximal aorta is passively stretched by the ejection flow and stores about 50% of the blood during systole. In diastole, the stretched wall recoils back and releases its potential energy to deliver blood flow to the coronary and peripheral arteries (28). The aortic wall movement is called the Windkessel function. This function avoids the excessive rise in ejection wave and pulse pressure and maintains the coronary perfusion. Histologically, this hemodynamic function is made capable by the effect of elastin, an elastic protein that is a major component of the proximal aortic wall. However, aging causes the proximal aortic stiffening characterized by severe structural remodeling of the arterial wall. Previous studies have reported that unlike the improvement of peripheral arterial function contributing to vascular stiffness, structural modification of the aortic wall is difficult to recover when exercise training was initiated in later life (29). However, in animal studies, repeated immersion has been reported to improve the aortic Windkessel and left ventricular functions (30). These authors described that water immersion leads to increased venous return due to elevations in hydrostatic pressure, cardiac output, and vascular wall stretch and shear stress. Another study also reported that elastin gene expression is enhanced by vascular wall stretching (31). In a human study, we found that lifelong Japanese female pearl divers who performed ~100–150 breath-holding free dives for up to 2 min at a time had lower aortic SBP, AIx, and heart-to-brachial PWV, which partly reflects the proximal aortic stiffness, when compared to inactive women of the same age (32). Likewise, in the present study, short-term aquatic exercise training in middle-aged and elderly subjects resulted in significant decreases in aortic PP and PPC, indicating the attenuated aortic pulsatility and the improvement of the proximal aortic Windkessel function. Collectively, increased venous return during water immersion may have a positive effect on the structure and function of the proximal aorta. On the other hand, Sherlock et al. reported that aortic SBP did not change significantly after a 10-week “shallow-water” aquatic exercise training (14). Although precise depth was not described, the lack of change in aortic SBP might be attributed to lower hydrostatic pressure which results in smaller increases in venous return and stroke volume compared with the present study.

Focusing on a single number as reflected by the ratio implies that information is lost. To overcome these simplifications, it is required to formulate alternative routes of data representation (17). Thus, in this study, we calculated PPC and AIxC by the polar coordinate description. Of note, AIxC decreased significantly after the intervention, although AIx did not change significantly. Parallel changes in aortic AP and PP might have masked the attenuated wave reflection with the aquatic exercise intervention as illustrated in Figure 1. Besides, change in aortic SBP was correlated with the corresponding change in AIxC but not AIx. The present study argues that these changes may not be identified by the conventional ratio-based indices. In this context, the newly introduced companion metric may provide better insight into underlying physiology. However, careful attention should be paid to the interpretation of the results of this index, as it has not yet been validated under a variety of physiological conditions.

### Study Limitation

Several methodological limitations should be mentioned. First, since aortic BP was estimated by a general transfer function from the brachial artery to the aorta, estimation error might not be equal among subjects. However, intra-individual change in estimates could be evaluated reliably. Second, arterial stiffness measurement using PWV may be prone to error due to inaccuracy of the arterial path length measurement. A sophisticated methodology such as MRI is required for evaluating the local proximal aortic stiffness response to the training intervention. Third, because of the nature of the field study, we could not control the characteristics of subjects (e.g., age, sex, and disease status). Furthermore, we did not have a sedentary control group, although we confirmed the excellent day-to-day reproducibility of key measurements. In addition, physiological exercise intensity (i.e., heart rate, oxygen consumption) during exercise training was not evaluated. These drawbacks should be overcome by future studies.

## Conclusion

In this study, we investigated whether head-out aquatic exercise training improves central aortic hemodynamics in middle-aged and elderly people. We found that head-out aquatic exercise training even at a low training frequency (once per week) may improve aortic hemodynamics. In particular, the reduction in aortic SBP was associated with the reduction in proximal aortic stiffness.

## Data Availability Statement

The original contributions presented in the study are included in the article/Supplementary Material, further inquiries can be directed to the corresponding author.

## Ethics Statement

The studies involving human participants were reviewed and approved by National Institute of Advanced Industrial Science and Technology Research Board. The patients/participants provided their written informed consent to participate in this study.

## Author Contributions

JS and MF decided on the conception, design of research, drafted the manuscript, and papered figures. MF, DH, TH, and JS performed the experiments, analyzed the data, and interpreted the results of the experiments. All authors edited and revised the manuscript and read and approved the final manuscript.

## Funding

This study was supported by special coordination funds of the Japanese. Ministry of Education, Culture, Sports, Science, and Technology (KAKENHI 17H02186 to JS) and the Descente and Ishimoto Memorial Foundation for the Promotion of Sports Science.

## Conflict of Interest

The authors declare that the research was conducted in the absence of any commercial or financial relationships that could be construed as a potential conflict of interest.

## Publisher's Note

All claims expressed in this article are solely those of the authors and do not necessarily represent those of their affiliated organizations, or those of the publisher, the editors and the reviewers. Any product that may be evaluated in this article, or claim that may be made by its manufacturer, is not guaranteed or endorsed by the publisher.

## References

[1] Lakatta EG Levy D. Arterial and cardiac aging: major shareholders in cardiovascular disease enterprises: part I: aging arteries: a “set up” for vascular disease. Circulation. (2003) 107:139–46. 10.1161/01.CIR.0000048892.83521.58 12515756

[2] Vlachopoulos C Aznaouridis K O'Rourke MF Safar ME Baou K Stefanadis C. Prediction of cardiovascular events and all-cause mortality with central haemodynamics: a systematic review and meta-analysis. Eur Heart J. (2010) 31:1865–71. 10.1093/eurheartj/ehq024 20197424

[3] Whelton PK He J Appel LJ Cutler JA Havas S Kotchen TA. Primary prevention of hypertension: clinical and public health advisory from the national high blood pressure education program. JAMA. (2002) 288:1882–8. 10.1001/jama.288.15.1882 12377087

[4] Haskell WL Lee I-M Pate RR Powell KE Blair SN Franklin BA. Physical activity and public health: updated recommendation for adults from the american college of sports medicine and the american heart association. Circulation. (2007) 116:1081–93. 10.1161/CIRCULATIONAHA.107.185649 17671237

[5] Khanam S Costarelli V. Attitudes towards health and exercise of overweight women. JR Soc Promot Health. (2008) 128:26–30. 10.1177/1466424007085225 18274327

[6] Tanaka H. Swimming exercise. Sports Med. (2009) 39:377–87. 10.2165/00007256-200939050-00004 19402742

[7] Waller B Ogonowska-Słodownik A Vitor M Rodionova K Lambeck J Heinonen A. The effect of aquatic exercise on physical functioning in the older adult: a systematic review with meta-analysis. Age Ageing. (2016) 45:593–601. 10.1093/ageing/afw102 27496935

[8] Alkatan M Machin DR Baker JR Akkari AS Park W Tanaka H. Effects of swimming and cycling exercise intervention on vascular function in patients with osteoarthritis. Am J Cardiol. (2016) 117:141–5. 10.1016/j.amjcard.2015.10.017 26541906

[9] Nualnim N Barnes JN Tarumi T Renzi CP Tanaka H. Comparison of central artery elasticity in swimmers, runners, and the sedentary. Am J Cardiol. (2011) 107:783–7. 10.1016/j.amjcard.2010.10.062 21247521

[10] Yuan W-X Liu H-B Gao F-S Wang Y-X Qin K-R. Effects of 8-week swimming training on carotid arterial stiffness and hemodynamics in young overweight adults. Biomed Eng Online. (2016) 15:673–84. 10.1186/s12938-016-0274-y 28155720 PMC5260035

[11] Wong A Kwak Y-S Scott SD Pekas EJ Son W-M Kim J-S. The effects of swimming training on arterial function, muscular strength, and cardiorespiratory capacity in postmenopausal women with stage 2 hypertension. Menopause. (2019) 26:653–8. 10.1097/GME.0000000000001288 30562322

[12] Cheung CP Coates AM Currie KD King TJ Mountjoy ML Burr JF. Examining the relationship between arterial stiffness and swim-training volume in elite aquatic athletes. Eur J Appl Physiol. (2021) 121:2635–45. 10.1007/s00421-021-04736-y 34132871

[13] Fukuie M Yamabe T Nomura Y Hashitomi T Maeda S Sugawara J. The effect of head-out aquatic exercise on arterial stiffness in middle-aged and elderly people. Pulse. (2019) 7:51–9. 10.1159/000498853 PMC11387882 39267880

[14] Sherlock L Fournier S DeVallance E Lee K Carte S Chantler P. Effects of shallow water aerobic exercise training on arterial stiffness and pulse wave analysis in older individuals. Int J Aquat Res Educ. (2014) 8:3. 10.25035/ijare.08.04.03

[15] Park SY Kwak YS Pekas EJ. Impacts of aquatic walking on arterial stiffness, exercise tolerance, and physical function in patients with peripheral artery disease: a randomized clinical trial. J Appl Physiol (1985). (2019) 127:940–9. 10.1152/japplphysiol.00209.2019 31369328

[16] Kerkhof PLM Peace RA Handly N. Ratiology and a complementary class of metrics for cardiovascular investigations. Physiology (Bethesda). (2019) 34:250–63. 10.1152/physiol.00056.2018 31165681

[17] Kerkhof PLM Konradi AO Shlyakhto EV Handly N Li JK. Polar coordinate description of blood pressure measurements and Implications for sex-specific and personalized analysiss. Annu Int Conf IEEE Eng Med Biol Soc. (2019) 2019:502–5. 10.1109/EMBC.2019.8857346 31945947

[18] Sato D Seko C Hashitomi T Sengoku Y Nomura T. Differential effects of water-based exercise on the cognitive function in independent elderly adults. Aging Clin Exp Res. (2015) 27:149–59. 10.1007/s40520-014-0252-9 24965855

[19] Raffaelli C Lanza M Zanolla L Zamparo P. Exercise intensity of head-out water-based activities (water fitness). Eur J Appl Physiol. (2010) 109:829–38. 10.1007/s00421-010-1419-5 20229021

[20] Tomoto T Sugawara J Hirasawa A Imai T Maeda S Ogoh S. Impact of short-term training camp on arterial stiffness in endurance runners. J Physiol Sci. (2015) 65:445–9. 10.1007/s12576-015-0383-6 26037815 PMC10717420

[21] Sugawara J Brothers RM Raven PB Okazaki K Ogoh S. Effect of systemic α1-adrenergic receptor blockade on central blood pressure response during exercise. J Physiol Sci. (2013) 63:389–93. 10.1007/s12576-013-0272-9 23771724 PMC10717366

[22] Murgo JP Westerhof N Giolma JP Altobelli SA. Aortic input impedance in normal man: relationship to pressure wave forms. Circulation. (1980) 62:105–16. 10.1161/01.CIR.62.1.105 7379273

[23] Motobe K Tomiyama H Koji Y Yambe M Gulinisa Z Arai T. Cut-off value of the ankle-brachial pressure index at which the accuracy of brachial-ankle pulse wave velocity measurement is diminished. Circulation J. (2005) 69:55–60. 10.1253/circj.69.55 15635203

[24] Vlachopoulos C Aznaouridis K Terentes-Printzios D Ioakeimidis N Stefanadis C. Prediction of cardiovascular events and all-cause mortality with brachial-ankle elasticity index: a systematic review and meta-analysis. Hypertension. (2012) 60:556–62. 10.1161/HYPERTENSIONAHA.112.194779 22733468

[25] Sugawara J Hayashi K Yokoi T Cortez-Cooper MY DeVan AE Anton MA. Brachial-ankle pulse wave velocity: an index of central arterial stiffness? J Hum Hypertens. (2005) 19:401–6. 10.1038/sj.jhh.1001838 15729378

[26] Tanaka H Munakata M Kawano Y Ohishi M Shoji T Sugawara J. Comparison between carotid-femoral and brachial-ankle pulse wave velocity as measures of arterial stiffness. J Hypertens. (2009) 27:2022–7. 10.1097/HJH.0b013e32832e94e7 19550355

[27] Sugawara J Hayashi K Tanaka H. Arterial path length estimation on brachial-ankle pulse wave velocity: validity of height-based formulas. J Hypertens. (2014) 32:881–9. 10.1097/HJH.0000000000000114 24609216

[28] Belz GG. Elastic properties and windkessel function of the human aorta. Cardiovasc Drugs Ther. (1995) 9:73–83. 10.1007/BF00877747 7786838

[29] Shibata S Levine BD. Effect of exercise training on biologic vascular age in healthy seniors. Am J Physiol Heart Circ Physiol. (2012) 302:H1340–6. 10.1152/ajpheart.00511.2011 22268113 PMC3311481

[30] Wang Y Bell A Wang H Kwabena B Fan S. The aortic windkessel property adaptation in whole-body immersion stressed mice. Angiol. (2018) 6:2. 10.4172/2329-9495.1000222

[31] Wanjare M Agarwal N Gerecht S. Biomechanical strain induces elastin and collagen production in human pluripotent stem cell-derived vascular smooth muscle cells. Am J Physiol Cell Physiol. (2015) 309:C271–281. 10.1152/ajpcell.00366.2014 26108668 PMC4537934

[32] Sugawara J. Effects of aquatic physical activity on proximal aortc function in middle-aged and elderly adults. Descente Sports Sci. (2018) 39:158–64.

