# Supplementary material for: Effect of Aquatic Exercise Training on Aortic Hemodynamics in Middle-Aged and Elderly Adults
Source: Front Cardiovasc Med. 2021 Nov 2;8:770519. doi: 10.3389/fcvm.2021.770519 (PMC8592941; doi:10.3389/fcvm.2021.770519)
Supplement: Supplementary file 1 [file Table_1.pdf]

Supplementary table 1: The correlation of changes in exercise trainig and aortic hemodynamic measurements

		ΔWeight	Baseline AoSBP	ΔAoSBP	ΔAoPP	ΔAoPPC	ΔbaPWV	ΔhaPWV	ΔAP	ΔAIx	ΔAIxC
Participation rate	*r*	-0.007	0.222	-0.163	0.125	-0.240	-0.132	-0.142	-0.374	-0.197	0.060
	P	0.975	0.308	0.458	0.571	0.269	0.548	0.519	0.078	0.368	0.785
ΔWeight	*r*		-0.321	0.027	0.050	0.012	-0.008	0.120	-0.191	-0.290	-0.014
	P		0.146	0.905	0.824	0.959	0.972	0.594	0.393	0.190	0.950
Baseline AoSBP	*r*			-0.350	-0.242	-0.325	-0.238	-0.192	0.082	0.232	-0.166
	P			0.102	0.266	0.130	0.274	0.379	0.711	0.286	0.448
ΔAoSBP	*r*				0.665	0.974	0.296	0.613	-0.375	0.056	0.632
	P				**0.001**	**<0.001**	0.170	**0.002**	0.078	0.800	**0.001**
ΔAoPP	*r*					0.481	0.107	0.270	-0.456	0.180	0.967
	P					**0.020**	0.628	0.212	**0.029**	0.410	**<0.001**
ΔAoPPC	*r*						0.314	0.627	-0.294	0.022	0.454
	P						0.145	**0.001**	0.173	0.921	**0.029**
ΔbaPWV	*r*							0.650	-0.095	-0.258	0.005
	P							**0.001**	0.665	0.234	0.981
ΔhaPWV	*r*								-0.329	-0.282	0.163
	P								0.126	0.192	0.456
ΔAP	*r*									0.700	-0.265
	P									**<0.001**	0.221
ΔAIx	*r*										0.413
	P										0.050

AoSBP, aortic systolic blood pressure; AoPP, aortic pulse pressure; AoPPC, the companion PP; baPWV, brachial-ankle pulse wave velocity; haPWV, heart-ankle pulse wave velocity; AP, augmentation preussure; AIx, augmentation index; AIxC, the companion AIx.
